# Terminating Cancer by Blocking VISTA as a Novel Immunotherapy: *Hasta la vista, baby*

**DOI:** 10.3389/fonc.2021.658488

**Published:** 2021-04-16

**Authors:** Ji-Eun Irene Yum, Young-Kwon Hong

**Affiliations:** Department of Surgery, Keck School of Medicine, University of Southern California, Los Angeles, CA, United States

**Keywords:** VISTA, immune-checkpoint, immunotherapy, cancer, tumor-microenvironment

## Abstract

VISTA is an up-and-coming immune checkpoint molecule that can become the target of new cancer immunotherapy treatments. Immune cells in the tumor microenvironment can largely influence the progression of cancer through inhibitory and stimulatory pathways. Indeed, VISTA is expressed on many immune cells, including T cells, myeloid-derived suppressor cells, tumor-associated macrophages, and dendritic cells. VISTA has predominantly been shown to act in an immune-suppressing manner that enables cancer progression. This review will delve into results from preclinical murine studies of anti-VISTA monoclonal antibody treatments, bring together recent studies that detect VISTA expression on immune cells from patient tumors of various cancers, and discuss ongoing clinical trials involving VISTA.

## Immune Checkpoint Therapy

Immune checkpoint molecules regulate the immune system’s inhibitory and stimulatory pathways in order to maintain homeostasis. Stimulatory checkpoint molecules can signal for an attack against infected cells or tumor cells, while inhibitory molecules can halt immune responses before they overreact to become self-harming. Immune cells must infiltrate the tumor microenvironment in order to keep tumor cells under control. However, their functions are often downregulated in the tumor microenvironment through immune-suppressing mechanisms that upregulate inhibitory pathways. Blocking the inhibitory checkpoint molecules can unleash the immune cells to proliferate better and fight against tumor cells. With this knowledge, immune checkpoint therapy is a growing form of treatment for an increasing number of cancer types.

Both CTLA-4 and PD-1 are inhibitory checkpoint molecules that limit activated T cells from their effector functions. The first immune checkpoint therapies to be developed were antibodies against CTLA-4 and, subsequently, antibodies against PD-1 (1). These treatments were game-changing by durably increasing cancer patients’ survival rates, but such benefits only applied to a fraction of patients and did not extend to all cancer types (2). Therefore, combination therapy was also proposed with both CTLA-4 and PD-1 blockade, which effectively increased response and survival rates but resulted in an increased risk of adverse events in patients (2).

New therapies targeting other checkpoint molecules that function in non-redundant pathways can fill the current gaps in the field. Each cancer type responds differently to the same treatments, and different patients with the same cancer type have varying treatment results. Hence, exploring the implications of the blockade or stimulation of various immune checkpoint molecules on each cancer subset can enable personalized therapeutic approaches with fewer side effects. Novel immune checkpoint therapies are under investigation in clinical trials, such as those targeting LAG-3, TIM-3, TIGIT, OX40, ICOS, VISTA, and many others (3). This paper will discuss the VISTA immune checkpoint molecule in detail, focusing on its cellular expression in the tumor microenvironment. VISTA can add to existing immunotherapy treatments by being a second line of treatment when CTLA-4 and PD-1 therapies are not effective. VISTA, unlike other negative checkpoint regulators that are expressed after T cell activation, is expressed on naïve T cells and enforces their quiescence in steady-state conditions. VISTA interaction can cause naïve T cells to undergo antigen-induced death or decreases in the TCR signaling and proliferation pathways (4). Therefore, VISTA blockade may help to expand tumor-antigen specific T cell numbers as well as to increase T cell activation in the tumor microenvironment. With notable expression on myeloid cells (5), anti-VISTA therapy can also extend beyond the T cell realm that the CTLA-4 and PD-1 therapies are centered around.

## Introduction to Vista

V-domain Ig suppressor of T cell activation (VISTA) has been identified in mice as an Ig superfamily inhibitory ligand with an extracellular domain bearing homology to PD-L1, a B7 family ligand (6). VISTA has various other aliases, including DD1α, PD-1H, Dies1, Gi24, and B7-H5. In humans, this gene is predominantly expressed in hematopoietic tissues or in tissues with large numbers of infiltrating leukocytes (5). In the hematopoietic compartment, VISTA has lower expression by T lymphocytes, while higher expression is seen in the myeloid compartment, notably by CD11b^high^ blood monocytes and dendritic cells (5).

VISTA-Ig fusion protein suppresses T cell proliferation *in vitro*, blocking the upregulation of early activation markers CD25 and CD69 without inducing apoptosis. VISTA-Ig also reduces cytokine production by T cells, including IL-10, TNF-α, and IFN-γ (5). This demonstrates the functionality of VISTA as a ligand. A different study found that VISTA can also function as a receptor through *in vitro* experiments where T cells from VISTA KO mice had increased proliferation compared to T cells from WT mice when pulsed with VISTA KO APCs (7). VISTA’s ability to act as both a ligand and a receptor is discussed further later in the review. Furthermore, VISTA monoclonal antibody (mAb) treatment enhances protective antitumor immunity in multiple murine cancer models by diminishing the suppressive nature of the tumor microenvironment (8). Upon such findings, VISTA has been proposed to be a promising new target for cancer immunotherapy.

Studies have already demonstrated how a monoclonal antibody treatment against VISTA could benefit the current repertoire of immune checkpoint therapies. In pancreatic cancer, which does not respond well to anti-PD-1 or anti-CTLA-4 therapy, PD-L1 and VISTA are expressed on different macrophage subsets in pancreatic tumor samples. This could indicate two separate inhibitory pathways capable of suppressing antitumor T cells (9). CD8+ T cells from human pancreatic tumors also face a greater inhibition of degranulation and cytokine production in the presence of VISTA-Ig than of PD-L1-Ig *in vitro* (9). Therefore, anti-VISTA therapy may be an effective immunotherapy treatment for pancreatic cancer patients by itself or in combination with anti-PD-1/PD-L1 therapy. In the case of metastatic melanoma, CTLA-4 and PD-1 inhibitors grant long-term survival to only a minority of patients due to innate and acquired resistance (10). Particularly, acquired resistance is frequent with anti-PD-1 inhibitors, and metastatic melanoma biopsies representing acquired resistance show an increased expression of intra-tumoral VISTA+ lymphocytes compared to pretreatment biopsies, pointing to the potential therapeutic implications of VISTA blockade (10). To understand how VISTA blockade can benefit patients as a cancer immune checkpoint therapy, we must analyze which cells express VISTA in the tumor microenvironment and the consequences of the expression.

## Vista Structure

A 162 amino acid extracellular domain, a 21 amino acid transmembrane domain, and a 96 amino acid cytoplasmic domain make up the human VISTA protein’s full structure. The cytoplasmic domain contains multiple casein kinase 2 and phosphokinase C phosphorylation sites but lacks any immunoreceptor tyrosine-based activation motifs. VISTA lacks an Ig-C domain, unlike other B7 family proteins that have been crystallized (11). VISTA’s extracellular domain has a β-sandwich conformation, with H-, A-, G-, F-, C-, and C’-strands making up the front face and A’-, B-, E-, D-, and C”-strands comprising the back face. The H-strand is unique to VISTA (12). There are three alpha-helices within this beta-sandwich conformation (11). Similar to other members in the immunoglobulin superfamily, VISTA has a disulfide bridging the B- and F-strands, a conserved tryptophan in the hydrophobic core, and a “Tyrosine corner” (12).

VISTA can be distinguished from the rest of the B7 family members with two additional disulfides: one that clamps the A’-strand to the H-strand and another that connects the CC’ loop to the F-strand (12). Furthermore, VISTA contains another 20 contiguous amino acids in the CC’ region, unlike other B7 family members. VISTA lacks the typical arginine-aspartate salt bridge present in other Ig-V proteins. Instead, it has an alternative salt bridge between Arg58 and Asp108 that reinforces the position of this unusually long CC’ region. Making up a large portion of VISTA’s extracellular domain, VISTA’s Ig-V-like domain of 149 amino acids is significantly longer than other V-set Ig domains. In addition to the long CC’ loop, the H-strand adds to the length as well. As a result of the disulfide tethering of the H-strand, the Ig domain has a short stalk of 9 amino acids, and VISTA has a C-terminal stalk emerging proximally to its N terminus rather than having the two termini distal to each other. This likely alters the angle from which the Ig-V-like domain is displayed from the cell surface, with restriction by the membrane’s close proximity. Deletion of the H-strand reduces T cell inhibition *in vitro*, whereas deletion of the H-strand and mutation of the disulfide bond’s cysteines leads to a complete loss of T cell inhibition. VISTA is also unique in the histidine content that makes up 8.6% of its extracellular residues, which is much higher than that of almost all other type I transmembrane extracellular domains. Strikingly, the histidine residues are completely concentrated in the CDR-proximal region. Therefore, they are oriented away from the membrane and able to interact with other receptors. In vitro and *in vivo* studies have shown that these histidine regions are critical for T cell inhibition, although mutating the histidines does not completely restore T cell proliferation (12). Overall, VISTA shows a relation to its B7 family members but has prominent differences as well.

## Vista Ligand/Receptor Interactions

**Figure 1:**
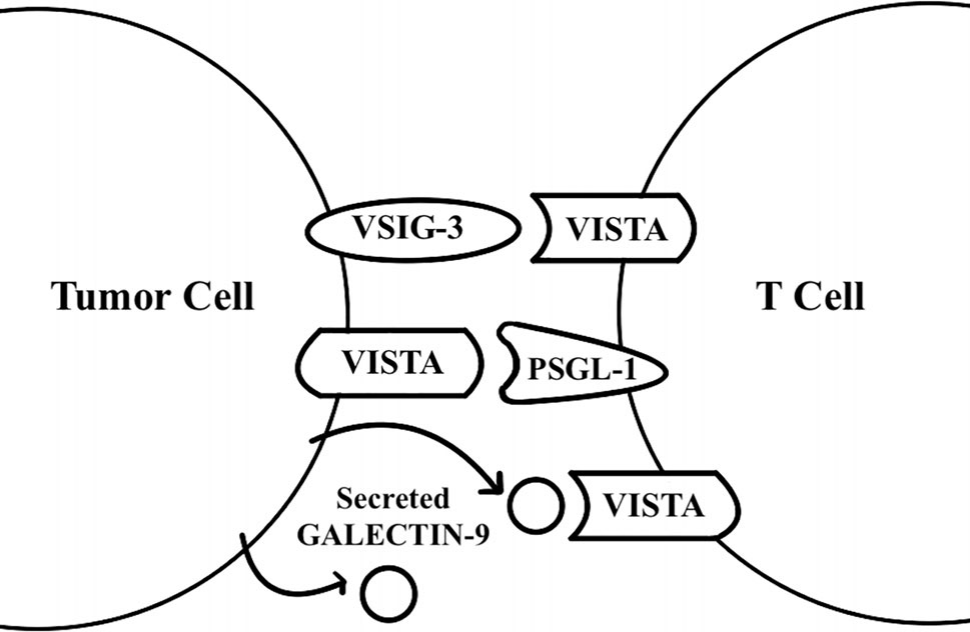
VISTA functions as a receptor and a ligand. VISTA was found to act as a receptor on T cells for the galectin 9 and VSIG-3 ligands, and as a ligand for the PSGL-1 receptor on T cells.

It is generally thought that VISTA may act as both a ligand and a receptor (**Figure 1**). VISTA has also been found to have homotypic interactions. Homophilic VISTA-VISTA binding facilitates intercellular interaction between apoptotic cells and macrophages for dead cell clearance (13). Further, an in-vitro study that treated WT T cells and VISTA KO T cells with VISTA-Ig protein demonstrates that VISTA KO T cell proliferation is less inhibited by VISTA-Ig protein. This shows that homophilic VISTA-VISTA interaction may also be important for T cell inhibition (13).

VISTA has been found to act as a receptor for the VSIG-3 ligand through binding assays (14). When human CD3+ T cells are treated with plate-coated VSIG-3, VSIG-3 inhibits anti-CD3 induced cytokine production, chemokine production, and T cell proliferation in a dose-dependent manner. Compared to T cells transfected with negative control siRNA, T cells transfected with VISTA siRNA have significantly greater cytokine secretion upon interaction with VSIG-3, demonstrating that VISTA acts as a receptor for VSIG-3 for the inhibition of T cells’ effector functions (14). The *in vivo* role of VSIG-3, however, remains to be determined. These findings on VSIG-3 and VISTA interaction do not explain how VISTA’s expression on other immune cells, such as Myeloid-derived suppressor cells (MDSCs) or antigen-presenting cell (APC), inhibits T cell proliferation and cytokine secretion. This is important because VISTA is more highly expressed on myeloid cells than on lymphocytes (5).

VISTA may preferentially engage its counter-receptor in more acidic environments because of the many histidine residues in VISTA’s extracellular domain that protonate at lower pH levels (15). VISTA-expressing cells suppress proliferation, IFN-γ production, and NF-κB phosphorylation by T cells at both pH 7.4 and acidic pH levels, with a significantly greater effect at acidic pH levels. Ligand-based receptor capture with VISTA and T cells at an acidic pH shows PSGL-1 receptor to be enriched. Gene deletion of this receptor significantly reduces VISTA binding to T cells *in vitro*, and ectopic expression of PSGL-1 on CHO cells enables VISTA binding at an acidic pH (15). These data support that VISTA may be a ligand to the PSGL-1 receptor. The in-vivo role of PSGL-1 with regards to VISTA, however, needs further studies.

Recently, galectin-9 has also been reported to be a ligand for VISTA. VISTA mediates galectin-9-induced downregulation of granzyme B release from T cells, trapping granzyme B inside the T cells and leading to apoptosis (16). There is a high likelihood for galectin-9 and soluble VISTA to form multi-protein agglomerates that engage with VISTA on the surface of T cells, which changes the plasma membrane potential and leads to granzyme B mediated self-killing of T cells (16). These findings come from studies of the interaction between THP-1 acute myeloid leukemia cells that heavily secrete galectin-9 and soluble VISTA, and Jurkat T cells, which highly express VISTA, so further research is needed to determine if the reported ligand-receptor interaction applies in a broader context. Future studies on VISTA’s homophilic interactions, VSIG-3, PSGL-1, galectin-9, and other counter-receptors that interact with VISTA would enable a better understanding of the VISTA molecule for the development of more effective therapies.

## Immune Cells in the Tumor Microenvironment

A healthy tumor microenvironment (TME) can help protect against tumorigenesis and invasion, while an unhealthy one can encourage further tumor growth (17). The TME is made up of various factors, such as the extracellular matrix, fibroblasts, adipose cells, neuroendocrine cells, immune cells, and the blood and lymphatic vascular networks (18). These components and their secretions can potentially provide support for tumor growth and impair host immune responses. Honing in on the immune cells, T cells, myeloid-derived suppressor cells (MDSCs), tumor-associated macrophages (TAMs), and dendritic cells (DCs) have a significant impact on the TME.

One common immunophenotype of the TME is the T cell infiltrated phenotype. A second phenotype of the TME is quite the opposite, with the exclusion of T cells from the tumor vicinity (19). Tumor-specific CD8+ T cells control tumors *via* immune effector functions. However, these T cells may be suppressed by immune system inhibitory players, such as Tregs, MDSCs, or TAMs, preventing them from carrying out effector functions, promoting tumor growth (19). Tumor-infiltrating Tregs exhibit augmented suppressive activity compared to Tregs isolated from peripheral blood and healthy tissues and a higher ratio of Treg/T effector cells within tumors is associated with poor prognosis in many cancers (20). Tregs may exert their suppressive activity through cytokines, immune checkpoint and inhibitory receptors, direct cytotoxicity, metabolic disruption of T effector cell activity, and induction of tolerogenic DCs (20).

Pathological activation of MDSCs results from low strength, persistent signals from tumors (21). MDSCs accumulate in both peripheral lymphoid organs and tumor tissues in tumor-bearing hosts. Those in the tumor are more immunosuppressive, potentially because of a higher monocytic-MDSC:polymorphonuclear-MDSC (M-MDSC : PMN-MDSC) proportion relative to the MDSCs in peripheral lymphoid organs and blood (22). Since PMN-MDSCs produce large amounts of ROS that are unstable and active temporarily, they need close contact with T cells that can only be provided by antigen-specific interaction. On the other hand, M-MDSCs produce high amounts of NO, Arg1, and inhibitory cytokines that are more stable; So, only proximity, but not antigen-specific contact to T cells, is necessary for their suppressive effects (22). MDSCs can induce or expand Tregs, deprive T cells of amino acids such as arginine and cysteine, nitrate TCR complexes –thus inhibiting T cell activation–, and interfere with T cell migration (23). Tumor-associated hypoxia increases PD-L1 expression by MDSCs as well, bringing inhibitory checkpoint receptors into action. MDSCs in the TME can rapidly differentiate into TAMs due to the hypoxic conditions (22).

TAMs closely resemble M2-polarized macrophages, which have anti-inflammatory, pro-tumorigenic properties (24). Their accumulation in tumors is associated with a worse clinical outcome, as they can cause immunosuppression, angiogenesis, and tumor progression. They can also modulate the tumor environment with growth factors and proteolytic enzymes to stimulate tumor metastasis. M2-macrophage-like TAMS have poor antigen-presenting capabilities and suppress T cells with IL-10 and TGF-β secretions (24). Tumor-infiltrating DCs tend to be associated with immunosuppression by low costimulatory molecule expression, decreased antigen cross-presentation, and high expression of regulatory molecules. Tumor cells and the TME can release factors that inhibit or reverse DC maturation, thereby abrogating their functions (25). As evidenced, a diverse set of immune cells in the TME can participate in functions that either enhance or suppress tumor growth.

## Vista Expression in the Tumor Microenvironment

### T Cells

Murine cancer models have been used to show how VISTA affects T cells of the TME. In murine melanoma models, VISTA antagonistic mAb treatment increases the percentage of tumor-specific CD4+ and CD8+ T cells within the tumor-infiltrating leukocytes (TILs) of the TME (8). The CD8+ T cells indicate tumor specificity by expressing CD44^high^ CD62L^low^ phenotypes and producing effector molecules like IFN-γ and granzyme B (8). Likewise, tumor-specific CD8+ T cells from a murine colon cancer model treated with anti-VISTA and anti-PD-L1 mAbs show enhanced inflammatory cytokine production as well as granzyme B production (26). These results point to VISTA’s inhibitory impact on T cell proliferation and effector function in the TME.

Another study using a mouse model of squamous cell carcinoma (SCC) found that anti-VISTA treatment converts non-functional resting memory and exhausted CD8+ T cells into functional effector CD8+ T cells (27). At an early time-point in this study, anti-VISTA monotherapy was shown to enhance the proportion of IFN-γ+TNF-α+ multifunctional CD8+ T cells. Again, these data convey that VISTA impedes T cell function, while VISTA blockade relieves such impediment. However, a decrease in Treg mediated immune suppression has been found to be crucial in fighting head and neck SCC, and anti-VISTA monotherapy alone is unable to increase the CD8+ T/Treg and CD4+ conventional T/Treg ratios in the TME of this SCC tumor; Combined CTLA-4 blockade and VISTA blockade can effectively increase these ratios in the TME (27). In certain cancers, VISTA blockade alone may not be enough but can work in synergy with other therapies.

VISTA expression by T cells has been confirmed in mouse and human studies. Using transgenic mice, CD4+ T cell expression of VISTA as a receptor has been found to be important for fighting tumor progression (7). In murine glioma models, VISTA KO mice treated with ionizing radiation have significantly longer survival than wild-type (WT) mice (7). CD4+ T cells play a major role in these results, as there is a notable brain-specific increase of IFN-γ+CD4+ T cells in the VISTA KO mice compared to the WT mice, while IFN-γ+CD8+ T cell numbers show no differences. Furthermore, CD4+ T cell depletion *in vivo* results in eliminating tumor resistance in VISTA KO mice treated with radiotherapy, whereas CD8+ T cell depletion has no effect.

Both CD4+ and CD8+ T cell expression of VISTA are detected after ipilimumab therapy is performed on prostate cancer patients who had no such expression observed before treatment – this indicates that T cells express VISTA in human TMEs as well (28). CD4+ and CD8+ T cells in the breast cancer TME also express VISTA (29), while only CD4+ T cells express VISTA in esophageal adenocarcinoma (EAC) tissue microarrays (30). In non-small cell lung cancer (NSCLC) tumor microarrays, VISTA expression is higher in T cells than in CD68+ macrophages (31). For human primary oral SCC, while there has been no significant difference found in overall survival rates between patients with high or low VISTA expression, VISTA^high^ CD8^low^ expression predicts significantly worse prognosis compared to any other subgroups combined: VISTA^low^ CD8^high^, VISTA^low^ CD8^low^, and VISTA^high^ CD8^high^ (32). This data analysis supports the importance of T cell infiltration into the TME for improved cancer prognosis and demonstrates a potential relationship between such infiltration and VISTA. Additional functional studies are needed to elucidate the consequences of increased VISTA expression by T cells in the TME.

As for Treg cells, in murine melanoma models, VISTA blockade significantly decreases the percentage of Tregs in the CD4+ T cell population within the tumor and tumor-draining lymph nodes (8). Such a decrease in the Treg population of the TME can contribute to greater CD4+ and CD8+ T cell activity against a tumor. Also, VISTA expression level is higher on tumor-infiltrating Tregs than on peripheral lymph node Tregs, which indicates VISTA may specifically influence TME-specific immune suppression (8). More research is needed to expand on VISTA’s role in Treg cells of the TME.

Overall, murine preclinical studies show that the expression of VISTA translates to CD4+ and CD8+ T cell suppression, while VISTA KO can lead to greater T cell proliferation and effector function. VISTA has also been shown to be expressed on CD4+ and CD8+ T cells in humans, but further research is needed to clarify the role of VISTA from T cells in the prognosis of different cancer types. There has been less research on VISTA’s influence on tumor-infiltrating Tregs, but existing research points to a decrease in Tregs with VISTA blockade, which contrasts with VISTA’s effect on CD4+ and CD8+ T cells. Further studies on the implications of VISTA KO on Tregs of the TME are crucial, as Tregs help mediate the line between a robust, helpful immune system and an auto-reactive, dangerous immune system.

### Myeloid-Derived Suppressor Cells

There is an increasing number of studies demonstrating VISTA expression by MDSCs in the TME. VISTA is highly expressed on tumor-infiltrating MDSCs in murine melanoma models, and anti-VISTA mAb treatment decreases MDSC percentages within the TILs of the TME (8). However, similar analysis in a murine bladder tumor model contrasts with the melanoma analysis, as MDSC percentages are not reduced in the tumors after the same treatment. A notable distinction is that the MDSC makeup of the bladder tumor model is primarily of the granulocytic type, while that of the melanoma model is primarily of the monocytic type (8).

VISTA+ cells among MDSCs have increased numbers in acute myeloid leukemia (AML) patients compared to healthy controls (33). VISTA expression on MDSCs from AML patients contributes to MDSC-mediated T cell suppression (33). The patients’ PBMC samples also show an interesting positive correlation between VISTA-expressing MDSCs and PD-1 expressing T cells, indicating a potential synergistic effect between the VISTA and PD-1 pathways in immune suppression. Another study analyzing cutaneous melanoma cases shows that high expression of CD33, an MDSC marker, correlates with pathological variables (34). These include Breslow thickness, ulceration, the likelihood of lymph node involvement, and an advanced AJCC stage. High VISTA expression is also associated with the same pathological variables. There is a significant correlation between the expression of MDSC marker CD33 and VISTA in the melanoma tissue, and double IF staining shows that CD33+ cells also express VISTA (34). Further, patients with low expression of CD33 and VISTA show better median overall survival than patients with high expression of just one of the two. These data suggest the potentially cancer-promoting effects of VISTA expression by MDSCs.

**Figure 2:**
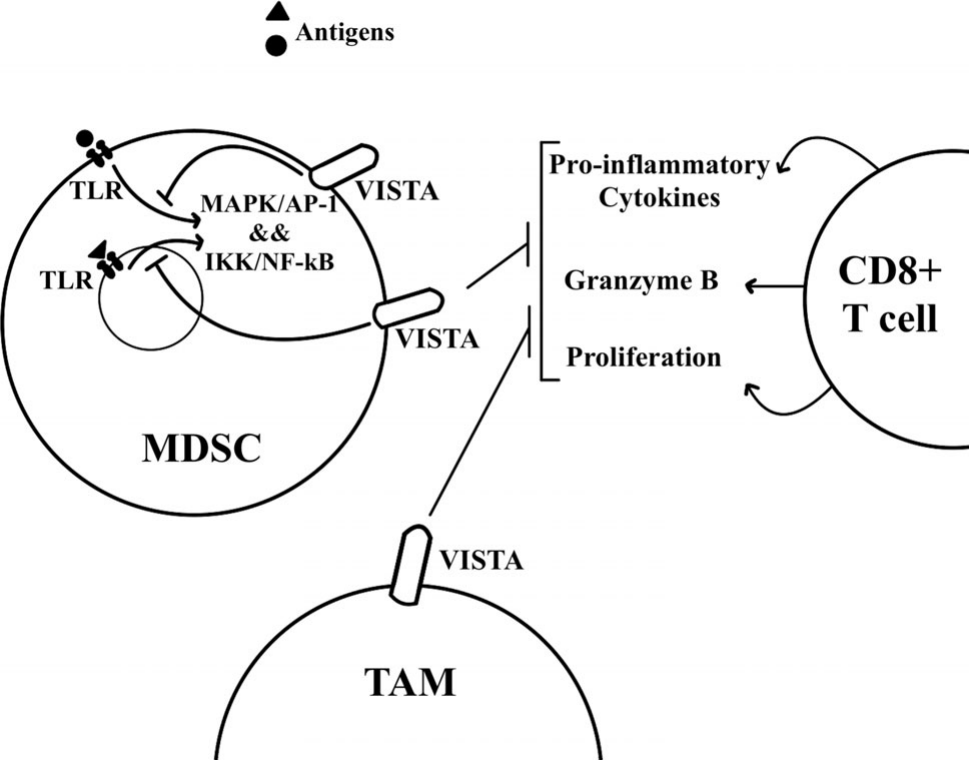
VISTA is expressed by MDSCs and TAMs in the tumor microenvironment. T cells undergo immunosuppression in the tumor microenvironment. MDSCs and TAMs can inhibit T cell proliferation and effector functions such as the secretion of inflammatory cytokines. VISTA on MDSCs has been found to inhibit TLR mediated activation of MAPK/AP-1 and IKK/NF-kB signaling cascades.

Mechanistically, VISTA transcription occurs in the hypoxic TME through HIF1A, which binds to the hypoxia response element in VISTA’s promoter (35). For isolated MDSCs from VISTA KO colon tumor-bearing mice, their absence of VISTA expression was found to have no effect on T cell proliferation and activation in a T cell suppression assay under normoxic conditions. Under hypoxic conditions, however, the absence of VISTA on MDSCs significantly increases T cell proliferation and activation. The same results can be seen with antibody blockade of VISTA, and such data support that hypoxia-induced VISTA expression contributes to MDSC mediated T cell suppression (35). The myeloid intrinsic function of VISTA works through the inhibition of TLR-mediated activation of MAPKs/AP-1 and IKK/NF-kB signaling cascades (**Figure 2**) through TRAF6 degradation (36). Both of these signaling cascades are important for the transcription of inflammatory cytokines and chemokines. VISTA blockade with TLR stimulation *in vitro* significantly increases the expression of IL-12 by M-MDSCs but not by PMN-MDSCs. Further, M-MDSC mediated inhibition of IFN-γ production by CD8+ T cells is reduced, but not PMN-MDSC mediated inhibition, indicating that PMN-MDSC may be resistant to inhibitors targeting VISTA (36). These data indicate the potential therapeutic benefits of VISTA blockade alongside TLR stimulation for reversing the suppressive functions of M-MDSCs. Primary oral squamous cell carcinoma (OSCC) tissue microarrays have been analyzed for further insights into the molecular regulation of VISTA (32). While VISTA expression is weak or negative in normal mucosa and in epithelial dysplasia, primary OSCC tissue shows high VISTA expression in tumor-infiltrating immune cells. VISTA expression is associated with MDSC markers, as well as with PD-L1 and CTLA-4. High VISTA expression also correlates with p-STAT3, for which activation prevents myeloid cell maturation, and IL-13Rα2, for which overexpression is associated with TGF-β1, a cytokine that plays a role in tumor metastasis and MDSC recruitment (32).

VISTA may play a role in the migration of MDSCs to the tumor microenvironment. A recent study using a mouse model of colon cancer shows that there are significantly fewer MDSCs in VISTA KO mice tumors than in WT mice tumors (37). Adoptive transfer of KO and WT MDSCs into WT tumor-bearing mice shows that KO donor MDSCs are much worse than WT donor MDSCs at infiltrating the tumor. An *in vitro* experiment further reveals that VISTA KO MDSCs are defective in migration towards chemokine CCL3, demonstrating that VISTA has an effect on MDSC chemotaxis (37). Overall, preclinical mouse models as well as human tumor tissue samples demonstrate the tumor-promoting function of VISTA expressing MDSCs, and various studies are underway to demonstrate further detail into the molecular mechanisms behind VISTA expression on MDSCs.

### Tumor-Associated Macrophages

TAMs also express VISTA and thereby suppress T cell attack against tumors (**Figure 2**), as can be seen through the analysis of tumor tissues from cancer patients. Indeed, VISTA expression in breast cancer and esophageal adenocarcinoma TME is mostly by TAMs (29, 30). TAMs express VISTA in resected colorectal carcinoma (CRC) tumors, where there is a significant correlation between the expression of VISTA and M2-macrophage signature genes (38). VISTA expression on M2 macrophages may mean VISTA is contributing to the anti-inflammatory, pro-tumorigenic properties of M2 macrophages. In fact, agonistic anti-VISTA treatment in-vitro has been shown to deter macrophages from committing to an M1 pro-inflammatory macrophage phenotype through reduced pro-inflammatory mediators and increased factors involved in macrophage regulatory activity and tolerance (39). Finally, the TME of immune checkpoint resistant pancreatic tumors is characterized by T cell exclusion alongside high levels of VISTA expressing macrophages (9). After ipilimumab (anti-CTLA-4) therapy, prostate cancer and metastatic prostate cancer patients have increased VISTA expression in post-treatment tumors (28). Specifically, the proportion of CD68+ macrophages with VISTA expression goes up fourfold. Post-treatment prostate tumor tissues also show a significant increase in Arg1 and CD163 expression, both genes associated with tumor-promoting M2-like macrophages. In vitro studies show plate-bound VISTA protein leads to a significant decrease in IFN-γ and TNF-α production by T cells from prostate cancer patients (28). However, pretreatment of patient monocytes with anti-VISTA antibodies before co-culture with patient T cells reverses this IFN-γ suppression. Therefore, anti-VISTA checkpoint therapy may further help ipilimumab-treated cancer patients by curbing T cell suppression mediated by VISTA expressing macrophages. Further, a pan-cancer study shows that for patients that received anti-PD-1/PD-L1 monotherapy, high expression of VISTA as well as CD68, a macrophage marker, is associated with significantly worse progression-free survival (40). There is likely a relationship between high VISTA expression and high CD68 expression here, as demonstrated by previously discussed studies.

Macrophages from VISTA KO mice have reduced consumption of CCL2 and CCL3 chemokines *in vitro* compared to those from WT mice (37). The CCL2 consumption reduction can be explained by a significant reduction of CCR2 surface expression on multiple myeloid subsets in the spleen. Similarly, a reduction of CCR5 (receptor for CCL3 and CCL5) surface expression also occurs, albeit only after additional treatment with CCL3 or CCL5 *in vitro*, denoting the inhibition of receptor recycling. Both CCR2 and CCR5 consumption of pro-inflammatory chemokines enable directed cell migration, and chemotaxis of VISTA KO macrophages to CCL3 and CCL5 are significantly disturbed relative to WT macrophages *in vitro*. This may explain the much lower frequencies of TAMs in tumor sections from VISTA KO than from WT tumors in a colon cancer mouse model (37). These experimental results demonstrate the potential of VISTA blockade to decrease TAM recruitment to the TME. Shown clearly through many studies, VISTA is expressed on TAMs in different cancer patients, and such expression may lead to immune system suppression.

### Dendritic Cells

In murine melanoma models, VISTA is highly expressed on tumor-infiltrating myeloid DCs (8). In a murine bladder tumor model, anti-VISTA mAb treatment enhanced the activation status of DCs by increasing their expression of MHCII and costimulatory CD80 as well as their production of IL-12 and TNF-α (8). Greater activation of the antigen-presenting DCs translates to increased stimulation of surrounding T cells that can then recognize and kill tumor cells. Similarly, DCs purified from the tumor tissues of TLR agonist and VISTA-blocking mAb treated mouse cancer models show increased IL-12 production and a decreased tendency to inhibit IFN-γ production by CD8+ T cells (36). VISTA blockade may thereby help increase T cell activity in the TME by shaping DC functions. Additional research can expand upon the cancer types in which DC expression of VISTA holds importance and upon the influence of VISTA expression by DCs compared to VISTA expression by the immunosuppressive cells of the TME.

## Vista in Relation to Combination Therapy

VISTA mAb treatment may be most effective in combination with other cancer therapies. A murine colon cancer model and melanoma model treated with anti-VISTA and anti-PD-L1 mAbs showed tumor regression and long-term survival, yet either mAb alone had less effective results (26). In addition, combination therapy performed on a murine melanoma model with anti-VISTA mAb and a peptide-based cancer vaccine showed significant survival benefits, while either treatment alone had little effect (8). Similarly, VISTA deficiency was not enough to significantly reduce tumor growth in a murine melanoma model, but the addition of a peptide vaccine inhibited tumor growth (41). Anti-VISTA monotherapy alone on a mouse model of SCC was unable to increase the CD8+ T/Treg and Tcon/Treg ratios in the TME of the SCC tumor, though combining CTLA-4 blockade and VISTA blockade was able to effectively increase these ratios (27). Finally, combined treatment with VISTA-blocking mAb and a TLR-agonistic vaccine led to tumor-free long-term survival for 50% of melanoma tumor-bearing mice, while either monotherapy by itself only had transient effects (36). In each of these studies, anti-VISTA therapy works synergistically with another treatment to reduce tumor growth or increase survival in a cancer model.

## The Potential Risks and Opposing Views of Vista Therapy

There is always the question in immunotherapy of how to treat cancer without exacerbating autoimmunity. Just as the existing anti-CTLA-4 and anti-PD-1 preclinical studies have demonstrated risks of autoimmunity from an overreactive immune system exerting inflammatory reactions, anti-VISTA therapy may potentially come with a comparable risk (13, 41). Blocking a non-redundant negative checkpoint molecule comes with the danger of an immune system that is less suppressed and therefore more likely to over-react. With higher frequencies of IFN-*y* secreting T cells, aged VISTA KO mice show chronic inflammation in multiple tissues, although they do not develop organ-specific autoimmune disease. VISTA knockout on both T and myeloid cells increases predisposition to the development of autoimmunity (41). Not surprisingly, there are various studies regarding how a VISTA agonist, rather than a VISTA antagonist, could be helpful for treating autoimmune pathologies or inflammatory diseases (42).

It is also important to note that VISTA therapy may specifically be helpful towards particular patient subsets. Different cancer types have varying degrees of correlation between VISTA expression in the TME and clinicopathological characteristics or patient outcomes. In contrast to various studies discussing VISTA as a tumor-promoting immune suppressor in the TME, there are also studies introducing VISTA expression by TILs as being positively correlated with survival, such as the studies with invasive ductal carcinoma (43) and EAC (30). There are also studies on the same cancer types that harbor disparate findings, such as those on VISTA expression in breast cancer (29, 43) and ovarian cancer (44, 45). Furthermore, this paper discusses VISTA expression by TILs, but we also must delve into the implications behind tumor cell expression of VISTA, as evidenced by studies with hepatocellular carcinoma (HCC) (46), CRC (38), breast cancer (29), gastric cancer (47), and ovarian cancer (45). A study on HCC shows that tumor cell expression of VISTA correlates with prolonged overall survival, and another on high grade serous ovarian carcinoma (HGSOC) shows that tumor cell expression of VISTA is associated with prolonged progression-free survival (45, 46). Therefore, in these cancer types, antagonistic VISTA therapy may be harmful to patients. VISTA has also been found to be expressed by endothelial cells in gastric cancer (47) and HGSOC (45). Taking such factors into account, further research is needed to clarify the roles of VISTA in different cancer types and stages, as well as in immune cells versus tumor cells.

## Clinical Trials

There are already ongoing clinical trials for anti-VISTA therapy. While an initial trial held by Janssen Pharmaceuticals was terminated (NCT02671955), Curis, Inc. is recruiting for another clinical trial (NCT04475523) with their CI-8993 anti-VISTA antibody, a human immunoglobulin G1κ monoclonal antibody targeting the VISTA ligand. This phase 1 study is enrolling about 50 patients with metastatic or unresectable solid tumor malignancy that is relapsed and/or refractory to prior therapy. There is also a PD-L1, PD-L2, and VISTA checkpoint antagonist called CA-170, an orally available small molecule, that is undergoing a phase I clinical trial by Curis, Inc. (NCT02812875), with results yet to be released. Adult patients with advanced solid tumors or lymphomas who have progressed or are not responsive to already available therapies were enrolled in this study.

## Conclusion and Future Directions

VISTA has the potential to become another target for cancer immune checkpoint therapy. More research still needs to be done to clarify the counter-receptors and ligands for VISTA, though PSGL-1, VSIG-3, and galectin-9 have already been shown to interact with VISTA, and homotypic VISTA interactions have also been supported. Preclinical studies in mice have demonstrated the immune-suppressing effects of VISTA in the TME. VISTA expression by MDSCs and TAMs has largely been shown to suppress T cell proliferation and cytokine production, promoting cancer progression (**Figure 2**). Further research is being conducted with human tumor specimens from various cancers, as more information is needed on which patient subsets could be helped or rather harmed by anti-VISTA therapy. Studies have also demonstrated potential for VISTA therapy in working synergistically with other treatments. With continued investigation, there is hope for antagonistic VISTA therapy as another immunotherapy treatment option.

## Author Contributions

J-EY wrote the article and Y-KH edited it. All authors contributed to the article and approved the submitted version.

## Funding

This study was supported by the National Cancer Institute and National Institutes of Health (R01CA250065 to YKH).

## Conflict of Interest

The authors declare that the research was conducted in the absence of any commercial or financial relationships that could be construed as a potential conflict of interest.
